# Computer vision quantification of whole-body Parkinsonian bradykinesia using a large multi-site population

**DOI:** 10.1038/s41531-023-00454-8

**Published:** 2023-01-27

**Authors:** Gareth Morinan, Yuriy Dushin, Grzegorz Sarapata, Samuel Rupprechter, Yuwei Peng, Christine Girges, Maricel Salazar, Catherine Milabo, Krista Sibley, Thomas Foltynie, Ioana Cociasu, Lucia Ricciardi, Fahd Baig, Francesca Morgante, Louise-Ann Leyland, Rimona S. Weil, Ro’ee Gilron, Jonathan O’Keeffe

**Affiliations:** 1 Machine Medicine Technologies Ltd., The Leather Market Unit 1.1.1 11/13 Weston Street, London, SE1 3ER UK; 2 Department of Clinical and Movement Neurosciences, Institute of Neurology, University College London, Queen Square, London, WC1N 3BG UK; 3 Neuroscience Research Centre, Molecular and Clinical Sciences Research Institute, St George’s, University of London, Cranmer Terrace, London, SW17 0RE UK; 4 Department of Clinical and Experimental Medicine, University of Messina, Messina, Italy, Via Consolare Valeria, 98165 Messina, Italy; 5 Dementia Research Center, Institute of Neurology, University College London, Queen Square, London, WC1N 3AR UK; 6 The Starr Lab, University of California San Francisco, 513 Parnassus Ave, HSE-823, San Francisco, CA 94143 USA

**Keywords:** Predictive markers, Parkinson's disease

## Abstract

Parkinson’s disease (PD) is a common neurological disorder, with bradykinesia being one of its cardinal features. Objective quantification of bradykinesia using computer vision has the potential to standardise decision-making, for patient treatment and clinical trials, while facilitating remote assessment. We utilised a dataset of part-3 MDS-UPDRS motor assessments, collected at four independent clinical and one research sites on two continents, to build computer-vision-based models capable of inferring the correct severity rating robustly and consistently across all identifiable subgroups of patients. These results contrast with previous work limited by small sample sizes and small numbers of sites. Our bradykinesia estimation corresponded well with clinician ratings (interclass correlation 0.74). This agreement was consistent across four clinical sites. This result demonstrates how such technology can be successfully deployed into existing clinical workflows, with consumer-grade smartphone or tablet devices, adding minimal equipment cost and time.

## Introduction

Bradykinesia, or slowness of movement, is one of the cardinal symptoms of Parkinson’s Disease (PD)^1^ and is a major determinant of patients’ quality of life^2^. The current gold standard for PD assessments is the Movement Disorder Society-sponsored revision of the Unified Parkinson’s Disease Rating Scale (MDS-UPDRS)^3^.

The third part of the MDS-UPDRS consists of 18 items that provide a measure of the severity of appendicular and axial motor signs. A patient’s motor impairment is measured on an ordinal 5-point scale from 0 to 4. This section is often used in clinical practice, especially for advanced therapies such as DBS (Deep-Brain Stimulation). It is also frequently a primary outcome measure in clinical trials^4^. Although health professionals are often highly trained, subjective appraisals are inevitable and can lead to undesirable consequences such as rater bias or rater drift^5–8^. Notwithstanding issues of subjectivity, a large-scale manual administration of the MDS-UPDRS requires a large number of clinicians and is therefore inherently unscalable and associated with high costs. An automated solution, that provided the same or similar information via algorithmic analysis would enable innovations such as large-scale remote clinical trials, comprising tens of thousands of subjects, or accelerated data-driven DBS programming through frequent patient re-assessment.

Wearable sensors have been extensively explored for disease management^9,10^, as well as quantification of upper-body bradykinesia^11^ and even whole-body bradykinesia^12,13^. Non-wearable sensors have demonstrated utility for disease management^14^ and quantification of upper-body bradykinesia^15^. However, all of these sensor-based approaches require additional dedicated hardware, which can substantially increase inconvenience and cost, largely preventing adoption in clinical practice.

The last decade has witnessed the widespread adoption of smartphone and tablet devices, capable of capturing high-quality videos^16^. Moreover, the video recording of neurological motor assessments is well established and has been common practice at many sites including those of the present study. Video-based approaches are therefore viable and considerably easier to adopt in the clinic^17^.

Several studies have explored using video data to measure bradykinesia in PD patients^18–21^. However, these studies addressed only upper-body bradykinesia and were limited by small samples (less than 150 patients, although often assessed multiple times) collected at a single clinical site. While upper- and lower-body bradykinesia has been explored^22^, this was also done with a limited sample from only two clinical sites in the same country.

Previous works demonstrate the potential of such technology, but not that it can generalise across multiple sites to a wider patient population. Given that training and assessment practices can vary between sites, multi-site validation is key to demonstrating efficacy for wider clinical use.

Here, we used a computer-vision-based approach to extract a small set of clinically interpretable objective metrics of bradykinesia in PD. We showed that the features could be used to train a model capable of estimating ten MDS-UPDRS bradykinesia ratings for each patient (left and right laterals, for the five limb-based bradykinesia items).

We extended previous research by (a) developing a system that delivered a single composite bradykinesia rating on a scale of 0–40, and (b) using more than 10 times the number of data points of previous vision-based studies, without the use of any manual filtering. These videos were recorded using consumer-grade hand-held devices, requiring only set-up (installation of KELVIN-CLINIC™ app from one of the app stores). Moreover, at four of five clinical sites, videos were recorded during routine MDS-UPDRS assessments.

## Results

### Multi-site patient population

**Fig. 1:**
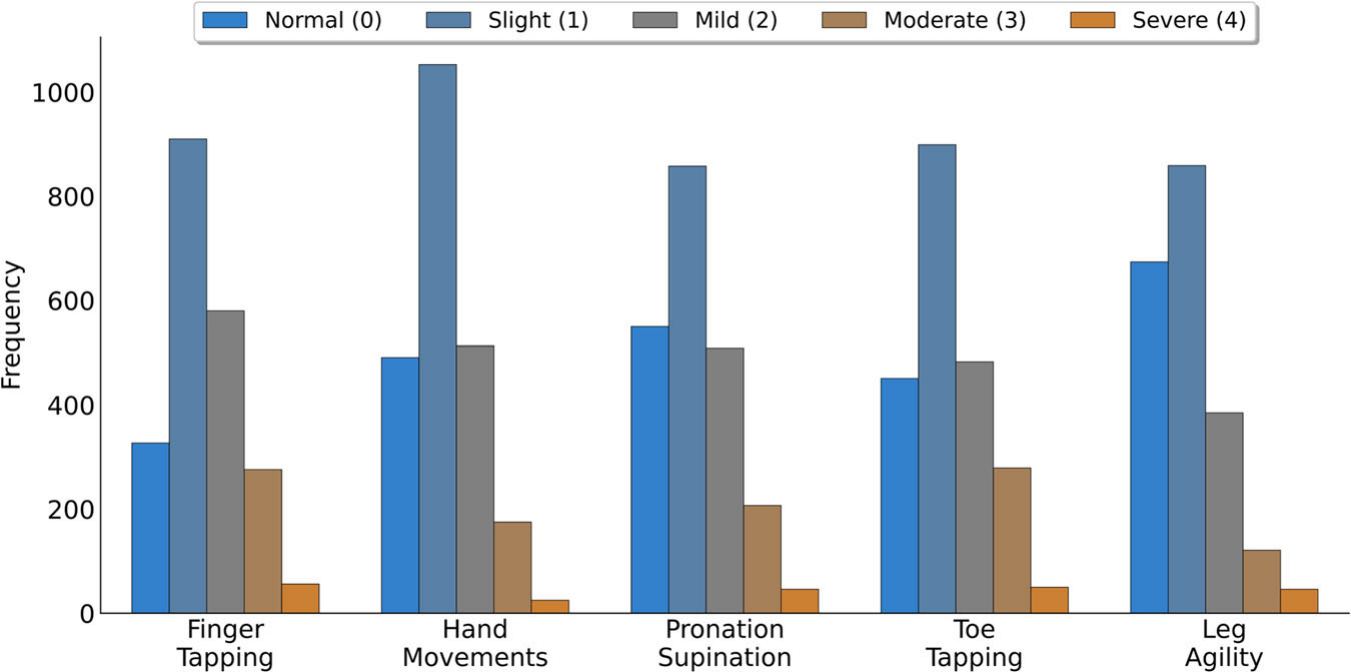
The distribution of MDS-UPDRS ratings for the five bradykinesia items. In each case the modal rating was Slight (1) and the least common rating Severe (4).

The dataset included videos of bradykinesia item examinations recorded as part of 1156 MDS-UPDRS assessments, of 628 separate PD patients (see Table 1). The vast majority of assessments contained ten ratings (left and right laterals for each of the five items), giving 10823 ratings in total, with an imbalance towards low ratings (see Fig. 1).

**Table 1:** Summary of the MDS-UPDRS assessment dataset.

	Assessments	Ratings	MDS-UPDRS part-3		Hoehn & Yahr Stage					
			Mean (SEM)	Q1–Q3	0	1	2	3	4	5
DCMN	649	6054	30.4 (0.7)	18–40	16	19	559	28	20	5
NRC	218	2031	35.3 (1.2)	22–45	1	2	156	43	14	1
DRC	132	1296	18.0 (1.0)	8–24	37	32	53	8	2	0
PDMDC	100	910	36.9 (1.3)	27–46	0	2	87	7	2	2
TSL	57	532	34.0 (2.6)	21–43	0	1	43	13	0	0
**All sites**	**1156**	**10823**	**30.6 (0.5)**	**18–41**	**54**	**56**	**898**	**99**	**38**	**8**

We show the mean and standard error of the mean (SEM), along with the lower and upper quartiles (Q1 and Q3), of the MDS-UPDRS part-3 total score. In addition, we show the breakdown of Hoehn & Yahr stage, as rated by the clinician conducting the MDS-UPDRS assessment.

Assessments were conducted by 15 different assessors, at 5 different sites, which are denoted; DCMN (Department of Clinical and Movement Neurosciences, Institute of Neurology, University College London), NRC (Neuroscience Research Centre, Molecular and Clinical Sciences Research Institute, St. George’s, University of London), DRC (Dementia Research Center, Institute of Neurology, University College London), PDMDC (Parkinson’s Disease and Movement Disorders Center, Baylor College of Medicine) and TSL (The Starr Lab, University of California San Francisco). Important to note that assessments at the DRC site were made by a research team, which doesn’t include clinicians.

A comparison of the sum of MDS-UPDRS scores could then be made, using the subset of 949 assessments for which all twenty ratings were available (five bradykinesia items, for both laterals, for both clinician and model ratings). From the subset of 949, 620 of the assessments (relating to 335 unique patients) had additional patient information; age, sex, disease duration, whether the patient had undergone deep-brain stimulation (DBS) surgery (see Table 2).

**Table 2:** Statistics summarising patient characteristics, for the 620 assessments where this additional information was available, broken down by clinical site.

	Age in years		Disease duration in years		Sex		Medication		DBS	
	Mean (SEM)	Q1–Q3	Mean (SEM)	Q1–Q3	Female	Male	Off	On	Yes	No
DCMN	59 (0.5)	54–66	7 (0.4)	3–9	86	209	179	116	34	261
NRC	61 (0.6)	57–66	12 (0.5)	9–14	40	116	42	114	117	39
DRC	66 (0.9)	59–71	5 (0.3)	3–7	41	45	8	78	1	85
PDMDC	63 (1.7)	58–71	9 (0.6)	6–10	15	22	30	7	0	37
TSL	52 (2.3)	40–63	10 (0.6)	7–12	9	37	10	36	46	0
**All sites**	**61 (0.4)**	**55–67**	**8 (0.2)**	**4–11**	**191**	**429**	**269**	**351**	**198**	**422**

For age and disease duration we show the mean and standard error of the mean (SEM) in years, as well as the lower to upper quartiles (Q1–Q3). We also show split by sex, medication (off-state vs on-state), and whether or not the patient had deep-brain stimulation at the time of assessment.

### Composite bradykinesia score

The composite bradykinesia (CB) scores (sum of items 3.4–3.8) obtained from the clinician (C-CB) and the models (M-CB) had a highly significant agreement (intraclass correlation (ICC) = 0.74, *p*-value < 0.001, *n* = 949). Four of the five sites had similar levels of agreement, as seen from the 95% confidence interval of the ICC including 0.74 (see Table 3). The one site that had lower agreement has two notable differences compared to the others. This group includes a higher proportion of medicated patients, and perhaps relatedly a lower mean disease severity at the time of assessment (see Table 2). Also, patients were assessed by non-clinician researchers, although the researchers had completed the MDS-UPDRS training. The lower level of agreement for this site can be explained by a limitation of the model in lower severity patients: MDS-UPDRS clinical rating of 0 can only be either matched or overestimated by the model creating a tendency for an overestimation in the composite score. However, other causes are possible such as higher levels of clinician ratings variability for on-medication patients^23^ or increased rater variability due to the non-clinical nature of the site^24^.

**Table 3:** Composite bradykinesia score results, broken down by site.

	Clinical score	Model score	Agreement		
	Mean (SEM)	Mean (SEM)	ICC	95% CI	n
DCMN	11.4 (0.3)	11.3 (0.3)	0.74	0.70–0.77	528
NRC	15.7 (0.6)	19.4 (0.5)	0.67	0.34–0.81	174
DRC	7.2 (0.5)	9.9 (0.4)	0.36	0.17–0.52	121
PDMDC	15.2 (0.7)	16.2 (0.7)	0.68	0.55–0.79	80
TSL	20.3 (1.0)	19.2 (1.1)	0.71	0.53–0.83	46
**All sites**	**12.4 (0.2)**	**13.4 (0.2)**	**0.74**	**0.70–0.77**	**949**

The mean and standard error of the mean (SEM) for the clinical and model scores, alongside the agreement between these scores, were measured by intraclass correlation (ICC), and the 95% confidence interval (CI) of the ICC.

**Fig. 2:**
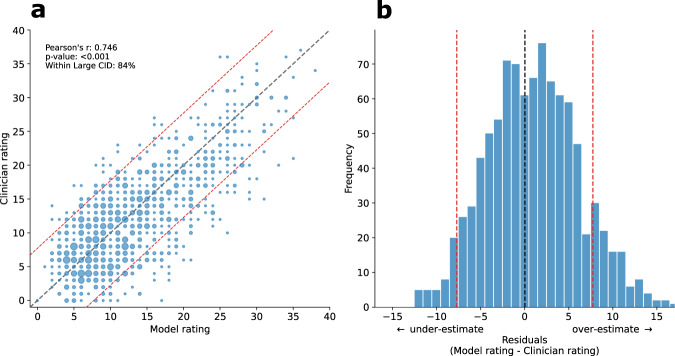
Composite bradykinesia score (sum of items 3.4–3.8) estimation results. **a** A scatterplot of composite bradykinesia scores for clinicians versus models, the size of dots corresponding to the number of patients with that combination of clinician and model ratings. **b** Distribution of residuals for the composite bradykinesia scores (*n* = 949). The mean was 0.97 which indicates that overall the models tended to slightly overestimate the composite bradykinesia score. Red dashed lines indicate the large clinically important difference (CID) band. For 84% of assessments, disagreement between clinician and the model fell below large CID.

**Fig. 3:**
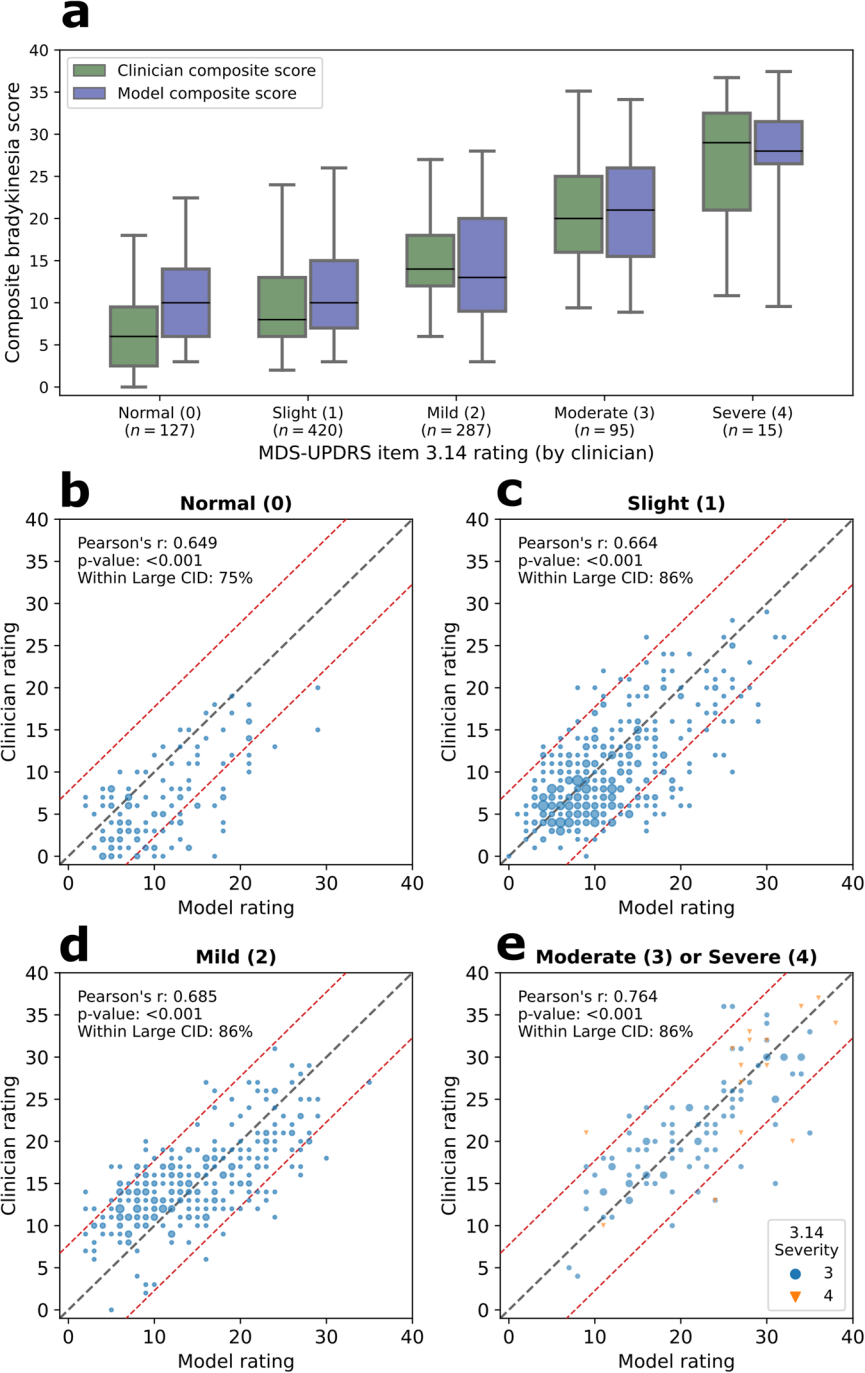
Composite bradykinesia scores (sum of items 3.4–3.8) split by body bradykinesia (item 3.14) scores. **a** Boxplots of composite scores indicate a similar distribution between the clinician and model scores for different levels of item 3.14. The box ranges from the first quartile to the third quartile of the distribution. The median is indicated by the center line. The whiskers indicate 2nd and 98th percentiles. (4 bottom panels) From top left to bottom right scatterplots of composite bradykinesia scores are shown for body bradykinesia scores of 0 (**b**), 1 (**c**), 2 (**d**), and 3&4 (**e**). Note that data corresponding to Moderate (3) and Severe (4), the minority classes, are binned together for analysis and presentation and marked by different colours and symbols. Across all severity scores, Pearson’s correlation coefficient is between 0.649 and 0.764, indicating a robust and consistent correspondence across all body bradykinesia severities. Red dashed lines indicate the large clinically important difference (CID) band.

The distribution of residuals of composite bradykinesia score, which had a mean of 0.97, indicates that overall our models tended to slightly overestimate MDS-UPDRS ratings (Fig. 2). Per-patient disagreements can occur, but large disagreements constitute a small proportion of assessments. For 84% of assessments, clinician and model disagreement fell below the large clinically important difference (CID)^25^ threshold. Please refer to Supplementary Notes 2 and 3 for a detailed residual analysis for demographic subgroups. The spread of clinician and model composite bradykinesia scores for each MDS-UPDRS item 3.14 score was similar (Fig. 3).

### Individual item classification

Overall, the MDS-UPDRS rating classifier achieved balanced accuracy of 45% (chance = 20%) and acceptable accuracy of 81%. The binary classifier, trained to distinguish between low and high severity ratings ({0, 1} vs {2, 3, 4}), had an accuracy of 75% and an area under the curve of the receiver operator characteristic (AUC-ROC) of 0.81. For both classifiers, the performance varied slightly between the five items (see Table 4).

**Table 4:** Performance of the classification models, broken down by each item.

	MDS-UPDRS classifier		Binary classification	
	Balanced Accuracy	Acceptable Accuracy	Accuracy	AUCROC
Finger Tapping	0.44	0.84	0.71	0.79
Hand Movements	0.43	0.86	0.74	0.81
Pronation-Supination	0.40	0.81	0.73	0.75
Toe Tapping	0.44	0.88	0.76	0.84
Leg Agility	0.52	0.91	0.80	0.86
**All items**	**0.45**	**0.86**	**0.75**	**0.81**

For the MDS-UPDRS rating classifier; balanced accuracy (average class recall) and acceptable accuracy (proportion of predictions within ±1). For the binary classifier; accuracy and area under the receiver operator characteristic curve (AUROC). For each of these evaluation metrics, there was a small variation between items, with pronation-supination tending to perform worse, and leg agility better.

**Fig. 4:**
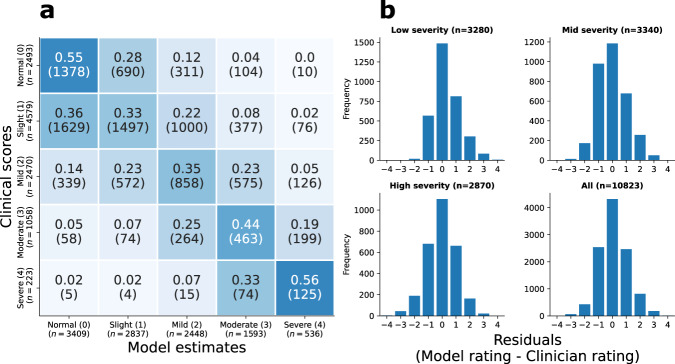
Summary of MDS-UPDRS rating classification model results. **a** Confusion matrix for all ratings for all five items. **b** Distribution of model residuals, separated by overall disease severity, as measured by the clinical composite bradykinesia score (low ≤ 8; 8 < mid ≤ 15; high > 15). For each of these severity groups, the modal residual was 0 (i.e. exact agreement).

**Fig. 5:**
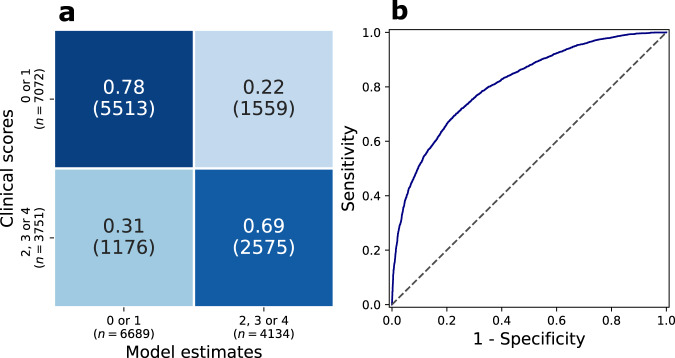
Summary of binary classification model results. **a** Confusion matrix of a binary classifier to distinguish low-mild versus moderate-high severity ratings ({0, 1} vs {2, 3, 4}), which has the accuracy of 75%. **b** Receiver operator characteristic curve of this classifier. The area under the curve is 0.81.

Figure 4 shows the confusion matrix across all five models (left panel), and residuals (right four panels), for composite scores for different composite severities. For low, mid, and high severities, and on aggregate, the modal residual was 0 (i.e. exact agreement between clinician and model). Figure 5 shows the confusion matrix and receiver operator characteristic curve of the binary classifier across all five items.

## Discussion

Markerless pose estimation was used to track patients’ movements during the bradykinesia examinations (finger tapping, hand movement, pronation-supination, toe tapping, leg agility) for the MDS-UPDRS part-3 motor assessment. The video data and associated clinical ratings were sourced from assessments at five independent sites, with no manual filtering of videos taking place. Features were extracted that capture key characteristics of impairment (such as velocity). A random forest model then utilised these computer-vision-based features, to objectively quantify a patient’s disease severity item by item. Ratings from these five items, measured separately on the left and right, were then summed to construct a composite bradykinesia score ranging from 0 to 40.

The model estimate of composite bradykinesia had high agreement with the clinician ratings (ICC = 0.74, *p*-value < 0.0001, *n* = 949). Examining the sites individually, we found that four of the five had 95% confidence intervals of ICC which included 0.74, indicating that the system had effectively generalised to the multi-site population. The site with lower agreement differed from the others in that it was a research site with lower severity patients, and the assessors were not PD clinicians. The lower level of agreement can be linked to the tendency of the models to slightly overestimate severity in milder patients.

The classifiers achieved a balanced accuracy of 45% and acceptable accuracy of 86%. Examining residuals by stratum of composite bradykinesia (low ≤8; 8 < mid ≤15; high >15) revealed that in all cases modal residual was 0, that is to say the exact agreement with the clinician assessor. A binary classifier, trained to distinguish between low and high severity ratings ({0, 1} vs {2, 3, 4}), had accuracy 75% accuracy and 0.81 AUC-ROC. Performance on this binary task was comparable to previous studies^20,26^.

New technology is unlikely to be embraced by clinical practitioners unless ease of use and generalisability can be demonstrated. Specialist sensors, both wearable^9^ and non-wearable^15^, can provide analytical value, but incur significant practical costs, for example, set-up time and other hardware management-associated issues, preventing adoption in the clinic. Computer-vision-based methods may be easier to use when deployed using smartphones or tablets, which are already widely used for multiple applications^17^. Previous work has demonstrated the efficacy of computer vision in quantifying whole-body bradykinesia^22^. However, in this study, a smaller sample of patients was assessed, with a majority of patients coming from a single clinical site. Thus the datasets were in all likelihood less heterogeneous as well as considerably smaller. In our study, we included more than 10 times the number of data points, collected across 5 different sites, and demonstrated that this technology can generalise across a wider PD patient population.

This work did not rely on any specific way of recording the data or any manual filtering for study inclusion. Data were collected during routine PD assessments, with clinicians conducting examinations according to their usual practices. Videos were recorded in a typical clinic/office setting, using standard consumer mobile devices. Our approach did not negatively impact the time taken to perform motor assessments. In contrast, due to automated data management, several clinicians reported a time-saving of 15–20 min per assessment, although this should be considered anecdotal. Obtaining the model scores after the assessments require manual annotation of the region of interest, which can be done through a web interface. Annotation and calculation of the model score usually take one minute per item. In summary, our results demonstrate that effective quantification of bradykinesia, through computer vision, can be deployed into clinical practice without adding friction to existing clinical workflows.

Martinez-Manzanera and colleagues found that four raters will agree on exact scores in only around 50% of cases^24^, and that three blinded raters have at least one disagreement in 40–50% of assessments and two disagreements in 1–5% of cases^22^ (implying a score difference of at least 2 between two of the rates). This phenomenon introduces potential complications for studies aiming to estimate the MDS-UPDRS ratings of clinicians. Additionally, it has been shown that two separate sets of clinical ratings, used to train a given classifier, can result in a substantial difference in classification performance^27^. Previous studies have employed the average of multiple raters^19,24^, or defined a successful model prediction as “agreement with any of the raters”^22^. Given our use of routinely collected clinical data, we had to rely on a single rater per video. However, we avoided “over-fitting” to a single opinion or local bias by using a dataset that is large and heterogeneous (over 10,000 ratings, from 15 different raters, at five different sites, across two continents).

Our results had a highly significant agreement for the composite bradykinesia score, and individual item ratings only diverged from clinical ratings by more than 2 points in 14% of cases, which shows performance approaching that of some clinical assessors. Furthermore, given the heterogeneity of opinions in our dataset, we would not expect, or desire, a model to always agree with the clinical rating. Indeed this would be an indicator of over-fitting. Rather, our models can be thought of as learning to emulate a weighted average of judgments, which may be more accurate and reproducible than any single clinician opinion, in keeping with literature on the wisdom of crowds^28^.

Motor dysfunction in PD is highly variable between patients and affects several aspects of movement^29^. For bradykinesia, the MDS-UPDRS lists speed, amplitude, hesitations, halts, and decrementing amplitude as cardinal criteria, but only provides subjective descriptions for how these should be used to rate severity (e.g. “slight slowing” for severity 1, and “mild slowing” for severity 2). Such imprecision in directions could itself account for a proportion of rater disagreements, although many other factors doubtless contribute. Despite this, severity scores are routinely used as a primary outcome measure of clinical trials testing the efficacy of pharmacological and surgical interventions^4^. Previous work has begun to explore objective metrics of bradykinesia criteria. Changes in the amplitude and velocities of actions might be related to different functional aspects of PD^30^, while alternative bradykinesia assessments have been developed to incorporate separate specific ratings for domains such as speed, amplitude, and rhythm^31,32^.

Our work, in addition to providing an objective composite bradykinesia score, can provide clinicians with detailed information about the characteristics of movements (speed, amplitude, hesitations, halts, decrementing amplitude) from the dozens of kinematic features that are inputted into the classification models. This information could be used by clinicians to improve decision-making and understanding of the complexities of motor impairments in PD.

Moreover, composite bradykinesia score can be combined with Gait and Arising from chair scores developed as a part of the same system^33,34^. A complete system can provide a richer and more complete patient assessment.

Our primary evaluation metric, the agreement between model and clinician as measured by the ICC, was significantly lower in one of the five sites. This could be explained by a significant proportion of medicated patients, a different population of raters, or this site examining only early-stage Parkinson’s disease patients. Therefore future work would focus on expanding the size of the dataset to gain a greater representation of different sites and the early-stage patient population, such that the system can fully generalise.

Estimating the composite bradykinesia score is an important step toward fully automated MDS UPRDS assessment. However, other important items such as speech, postural stability, and tremor are still required to complete the picture. These items have the potential to be estimated through a similar framework and the development of such models will be our future work.

The classification models relied upon manual feature engineering (shallow features), guided by clinical understanding. Deep learning can perform automated data-driven extraction of features (deep features), although potentially at the cost of losing some interpretability. Deep learning models have been shown to be effective for quantifying bradykinesia accurately^35,36^. A system that combined both shallow and deep learning might be hypothesised to be optimal, in terms of both accuracy and interpretability, although this remains to be shown.

The analysis in this study was 2D vision-based, relying on 2D pose estimation. Inevitably some information relating to the third dimension was lost. Although dedicated hardware could be used to address this, for example, a Kinect sensor also provides an estimate of depth^15^, this would discard the convenience of already-available consumer devices for data collection. Encouragingly, studies have shown that 3D pose estimation can be done using monocular images^37,38^, and this has been applied to quantifying gait impairment in PD with higher model accuracy reported when using 3D pose compared to 2D pose^39^. This suggests that future work focusing on bradykinesia could benefit from 3D information. Thanks to improvements in both 3D pose estimation techniques and device sensors, tablets and smartphones increasingly include dedicated depth sensors such as LiDAR, which presents the possibility of capturing depth data without adding additional complexity to the assessment.

The possibility of remote assessments based on smartphones is receiving increasing attention^22,40–42^. Future work will include validating automated quantification of whole-body bradykinesia in the remote context, with patients using a mobile app to record video assessments within their home, then those videos and automated bradykinesia scores being delivered to clinicians via cloud computing infrastructure.

We presented a computer-vision-based method capable of quantifying whole-body bradykinesia from video data collected during routine MDS-UPDRS assessments at multiple sites, using widely available consumer-grade mobile devices and without the need for manual filtering. While the results are highly statistically significant, and comparable to previous studies in the field, future improvements in hardware and increases in the quantity of data available for model training, and likely to result in further improvements in performance.

This system has the potential to standardise bradykinesia assessment across sites, locally or remotely, and to make clinical data acquisition at scale a realistic possibility for both clinical care and research.

## Methods

### Subjects and assessments

MDS-UPDRS assessments of PD patients were conducted by examiners at five movement disorders centres in the United Kingdom and the United States. Symptom severity for each of the five bradykinesia items was measured on an ordinal 5-point scale from 0 to 4. Video recordings and ratings were captured through KELVIN™, a video-based motor assessment platform developed by Machine Medicine Technologies^17,43^, which has underpinned previous work on other MDS-UPDRS items^33,34^. Videos were recorded across a wide range of disease severity, in different medication states (ON, OFF, wearing off), and deep-brain stimulation states (ON stimulation, OFF stimulation), using consumer cameras integrated within mobile devices or tablets. The majority (approx. 90%) of the videos were recorded at 1080 × 1920 resolution and 29.97 framerate, a capability commonly available for most modern mobile devices.

Although videos were automatically filtered using criteria such as minimum length and minimum frame rate, no manual selection of videos took place, and the data thus reflects the current state of routinely collected clinical data at these sites. The only instructions given to clinicians were to recommend the use of a tripod, and to keep the patient fully visible and centred within the video frame at all times. Only one frontal view was captured during the assessment.

In this study, we focused on the five MDS-UPDRS items assessing the severity of a patient’s bradykinesia symptoms: items 3.4 (finger tapping), 3.5 (hand movement), 3.6 (pronation-supination), 3.7 (toe tapping) and 3.8 (leg agility). It is worth noting that while other items such as 3.10 (gait) can also be affected by bradykinesia, for this work we only included items primarily designed to assess the severity of bradykinesia. Although item 3.14 (global spontaneity of movement or body bradykinesia) also focuses on bradykinesia, it is a summary rating based on all observations during the assessment rather than on a single item.

### Pose estimation and signals

**Fig. 6:**
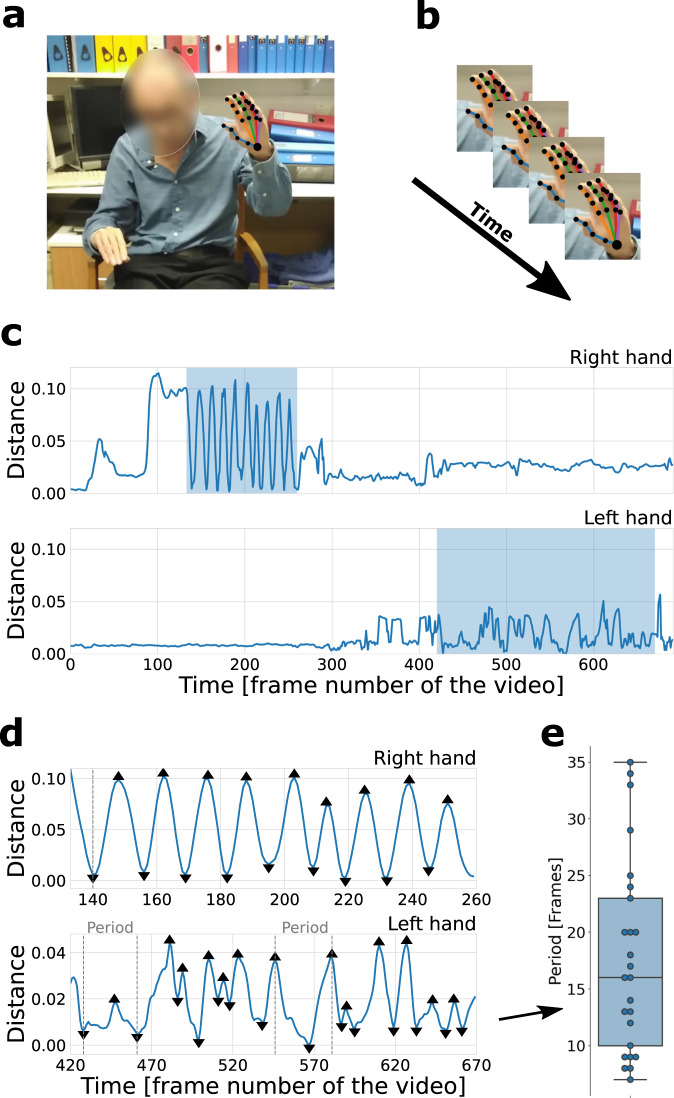
Feature extraction overview. **a** The deep learning library OpenPose^44^ was used to extract 25 body and 21 hand key points from each frame of the video. The photograph is published with written consent from the patient. **b** Coordinates of the key points across the frames were used to construct time-series signals. **c** An example of finger-tapping signals (i.e. Euclidean distance between index finger tip and thumb-tip key points) for right (top) and left (bottom) hand. In this case, the right hand received a low severity score of 1, while the left hand received a high severity score of 4. The highlighted regions depict the regions of interest (ROIs); i.e. when the action was performed. **d** Detected peaks and troughs on the signals of the two ROIs for the right hand (top) and left hand (bottom). Features were constructed from these signals. The time between peaks corresponds to the time between successive finger taps. **e** The distribution of periods (in number of frames between consecutive peaks and troughs) is extracted from the lower panel (left-hand signal) of (D). The box ranges from the first quartile (Q1) to the third quartile (Q3) of the distribution, the median is indicated by the center line, the whiskers indicate the distance 1.5*IQR (interquantile range) below Q1 and above Q3. One of the features, range of period between actions, is calculated as maximum minus minimum period.

The deep learning library OpenPose^44^ was used to extract 25 body and 21 hand key-point coordinates on each frame (see Fig. 6). Prior to pose estimation, all videos were rescaled to 640 × 1138 resolution. Experimentation with full and downscaled versions didn’t show any significant changes in the performance of the system. For each bradykinesia item, signals based on key-points relevant to the appropriate action were constructed. These signals were normalised using the patient’s estimated-standing height (height estimation model was developed during previous research^33,34^, see Supplementary Note 4 for details), with the exception of the pronation-supination signal which was an angular measure and thus much less dependent on the distance between the patient and the camera.

A peak detection algorithm was used to identify local maxima (peaks) and minima (troughs), which typically correspond to the start and midpoint of a periodic action. For example, as the finger tapping signal was based on the distance between thumb and index finger tip, a peak would correspond to the two fingers being maximally apart, and a trough would correspond to the two fingers touching. Videos were annotated manually with regions of interest (ROIs); i.e. the videos were labelled with sections during which a particular action was performed using a specific body part. For example, finger-tapping videos would usually contain two ROIs, corresponding to the sections of the video in which the patient performed the action using their left and right hand. The time-series signals were cropped to these ROIs, with features then being extracted from these cropped signals.

### Signal computations

For each of the MDS-UPDRS items (see Table 5) a time-series signal was constructed to capture the action being carried out.

**Table 5:** Instructions given to patients for each of the five main bradykinesia items in the MDS-UPDRS^3^.

MDS-UPDRS item	Instructions to patient
Finger Tapping	Tap the index finger on the thumb 10 times as quickly and as big as possible.
Hand Movement	Open the hand 10 times as fully and as quickly as possible.
Pronation-Supination	Extend the arm out in front of the body with the palms down; then to turn the palm up and down alternately 10 times as fast and as fully as possible.
Toe Tapping	Place the heel on the ground in a comfortable position and then tap the toes 10 times as big and as fast as possible.
Leg Agility	Place the foot on the ground in a comfortable position and then raise and stomp the foot on the ground 10 times as high and as fast as possible.

These five signals are defined in Table 6 using the following notation:

- *x*_*b*_(*i*), *y*_*b*_(*i*), **P**_*b*_(*i*) are the x-coordinate, y-coorindate and 2D positional vector of the *i*^*th*^ body key-point respectively.
- *x*_*h*_(*i*), *y*_*h*_(*i*), **P**_*h*_(*i*) are the x-coordinate, y-coorindate and 2D positional vector of the *i*^*th*^ hand keypoint.
- **V**_*h*_(*i, j*) is the vector drawn from the *i*^*th*^ hand keypoint to the *j*^*th*^ hand keypoint.
- **H** is the estimated-standing height, which is used to normalise pixel distances to account for patients of different heights and at different distance to camera.

**Table 6:** Signals constructed from key-points and used for feature extraction for each of the five bradykinesia items.

MDS-UPDRS item	Time-series signal	Formula
Finger Tapping	Euclidean distance between the thumb-tip key-point and the index finger tip key-point, measured in units of estimated-standing height.	${\textstyle{\overrightarrow{\left| {{\left. {{{{\mathbf{P}}}}_{h(4)}{{{\mathbf{P}}}}_{h(8)}} \right)} } \right|} \over {{{\mathrm{H}}}}}}$
Hand Movement	The area of the convex hull (ACH) of the four finger tips key-points and the palm key-point, measured in units of estimated-standing-height squared (H^2^).	${\textstyle{{{{{\mathrm{ACH}}}}\left( {{{{\mathbf{P}}}}_{h(i)}:i \in \left( {0,8,12,16,20} \right)} \right)} \over {{{{\mathrm{H}}}}^2}}}$
Pronation-Supination	The angular velocity of the vector from the thumb-tip key-point to the little-finger-tip key-points, measured in degrees per frame.	$\angle \left( {{{{\mathbf{V}}}}_{h(4,20)}^{t - 1},{{{\mathbf{V}}}}_{h(4,20)}^t} \right)$
Toe Tapping	The vertical distance between the small toe and the neck, measured in units of estimated-standing height.	$${\textstyle{{\left( {{{{\mathbf{y}}}}_{b(i)} - {{{\mathbf{y}}}}_{b(1)}} \right)} \over {{{\mathrm{H}}}}}},\quad i = \left\{ {\begin{array}{*{20}{l}} {20} \hfill & {{{{\mathrm{left}}}}} \hfill \\ {23} \hfill & {{{{\mathrm{right}}}}} \hfill \end{array}} \right.$$
Leg Agility	The Euclidean distance between the knee key-point and the neck key-point, measured in units of estimated-standing height.	$${\textstyle{\overrightarrow{| {{{{\mathbf{P}}}}_{b(i)}{{{\mathbf{P}}}}_{b(1)}} |} \over {{{\mathrm{H}}}}}},\quad i = \left\{ {\begin{array}{*{20}{l}} {13} \hfill & {{{{\mathrm{left}}}}} \hfill \\ {10} \hfill & {{{{\mathrm{right}}}}} \hfill \end{array}} \right.$$

### Peak/trough detection

The *find peaks* function from the Python library Scipy, was run on each signal to identify local maxima (peaks) and run on the negative of each signal to identify local minima (troughs). The performance of this function depends on a number of parameters, such as the minimum interval between consecutive peaks and the minimum height of a peak. In order to calibrate the function for use on the extracted signals a grid search of parameter values was run, separately for each bradykinesia item, with the chosen sets (see Table 7) being those that minimised the mean squared error between estimated and manually labelled frequency for a given item.

**Table 7:** Chosen find_peaks parameter set for each bradykinesia item.

Item	height	threshold	distance	width	rel_height	prominence	wlen
Finger Tapping	None	None	4	0.5	0.75	0.3	15
Hand Movements	MA:10	None	5	4	0.5	1	60
Pronation-Supination	MA:35	(0,1.5)	5	3	0.5	0.5	10
Toe Tapping	None	None	None	None	1	0.3	10
Leg Agility	None	None	None	None	1	0.7	60

MA:*x* denotes the moving average of the signal computed with a window size of *x* frames.

### Feature extraction

**Fig. 7:**
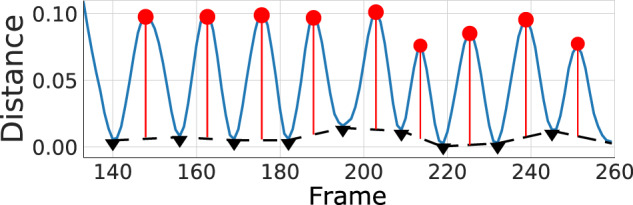
Peak detection illustration. The amplitude of an action is measured as the vertical distance from a peak (red dot) to the linear interpolation between troughs (black triangles).
- **VEL()**: the function that returns the mean absolute first difference of a time-series (referred to below as velocities).
- **V**: **VEL()** applied to each element of S*: **V** = (VEL(*S*_i_*) : *i ∈* [1*, ..., m* + *k* + 1])
- **INV()**: the function that returns the percentage of points in a time-series that need to be inverted in order to create a monotonic time-series.
- **I**: **INV()** applied to each element of S*: **I** = (INV(*S*_i_*) : *i ∈* [1*, ..., m* + *k* + 1]).

For each of the five bradykinesia items, the same 11 features were extracted from the relevant time-series signal. These eleven features are defined in Table 8 using the following notation:

- **s** = (*s*_1_, ..., *s*_*n*_): the y-values of the time-series, where x-values are frame numbers (see Table 6).
- **s**′: “Absolute velocity”, which is the absolute first difference of **s**.
- **s**″: “Absolute acceleration”, which is the absolute first difference of **s**′.
- **s**″′: “Absolute jerk”, which is the absolute first difference of **s**″.
- **p** = (*p*_1_, ..., *p*_*m*_): the list of frame numbers of each local maxima (peaks).
- **t** = (*t*_1_, ..., *t*_*k*_): the list of frame numbers of each local minima (troughs).
- **d** = (*d*_1_, ..., *d*_*m*+*k−*2_): the concatenation of the list of differences between consecutive peaks, (*p*_*i*+1_ − *p*_*i*_ : *i ∈* [1*, ..., m −* 1]), and the list of differences between consecutive troughs, (*t*_*i*+1_ − *t*_*i*_ : *i ∈* [1*, ..., k −* 1]).
- **o**: the ordered version of the concatenation of the two lists **p** and **t**.
- o*: the concatenation of the three lists (1), **o** and (*n*).
- **S**: the list of subsets of time-series based on **o**; ((*s*_*oi*_, ..., *s*_*oi*+1_) for *i ∈* [1, ..., *m* + *k −* 1])).
- S*: the list of subsets of time-series based on o*; ((*s*_o*i_, ..., *s*_o*i+1_) for *i ∈* [1, ..., *m* + *k* + 1])).
- **f** = (*f*_1_, ..., *f*_*m*+*k−*2_): the frequency estimates at each peak and trough (measured in Hz); (${\textstyle{{FPS} \over {d_i}}}$: *i ∈* [1*, ..., m* + *k −* 2]), where *FPS* is frames per second of the video.
- **a** = (*a*_1_, ..., *a*_*m*_): the list of amplitudes of each peak, measured as the difference between the y-value at that peak and the y-value of the lower bound at that peak, where the lower bound is the line resulting from linearly interpolating between consecutive troughs (see Fig. 7).

**Table 8:** Features extracted from the time-series signals (see Table 6).

Feature	Description	Formula	Group
*MeanFreq*	Mean frequency of actions	mean(f)	Speed
*CovarFreq*	Coefficient of variation of the frequency of actions	${\textstyle{{{{{\mathrm{std}}}}({{{\mathrm{f}}}})} \over {{{{\mathrm{mean}}}}({{{\mathrm{f}}}})}}}$	Speed
*MeanVel*	The average of the velocities between peaks and troughs	mean(V)	Speed
*CovarVel*	Coefficient of variation of the velocities between peaks and troughs	${\textstyle{{{{{\mathrm{std}}}}({{{\mathrm{V}}}})} \over {{{{\mathrm{mean}}}}({{{\mathrm{V}}}})}}}$	Speed
*MeanAmp*	Mean of amplitude of actions	mean(a)	Amplitude
*CovarAmp*	Coefficient of variation of the amplitude of actions	${\textstyle{{{{{\mathrm{std}}}}({{{\mathrm{a}}}})} \over {{{{\mathrm{mean}}}}({{{\mathrm{a}}}})}}}$	Amplitude
*PeriodRange*	The range of period of actions	range(d)	Hesitations
*PrcInv*	Average of the rate of inversions between peaks and troughs	mean(I)	Hesitations
*Roughness*	The median of the absolute jerk divided by absolute acceleration	${{{\mathrm{median}}}}\left( {{\textstyle{{s^{\prime\prime \prime}} \over {s^{\prime\prime}}}}} \right)$	Hesitations
*DiffAmp*	Percentage change between *MeanAmp* of first third of peaks (denoted A^*T*1^) and the last third of peaks (denoted A^*T*3^)	${\textstyle{{{{{\mathrm{A}}}}^{T3} - {{{\mathrm{A}}}}^{T1}} \over {{{{\mathrm{A}}}}^{T1}}}}$	Decrementing signal
*DiffVel*	Percentage change between *MeanVel* of the first third of peaks (denoted *W*^*T*1^) and the last third of peaks (denoted *W*^*T*3^)	${\textstyle{{{{{\mathrm{W}}}}^{T3} - {{{\mathrm{W}}}}^{T1}} \over {{{{\mathrm{W}}}}^{T1}}}}$	Decrementing signal

The same 11 features were used for each bradykinesia classification model.

These features were intended to capture the key characteristics of the movement, and could roughly be grouped into the four main aspects of impairment described by the MDS-UPDRS; speed, amplitude, hesitations and halts, and decrementing signal.

For illustration, patients with more severe impairment were expected to slow down and perform fewer actions per second, as well as executing the actions less smoothly. The amplitude of actions was also expected to decrease with impairment severity. For example, during finger tapping, severely impaired patients would not be able to vary the distance between their thumb and index finger, compared to less impaired patients.

### Individual item models

To estimate individual MDS-UPDRS ratings, an ordinal classification system^45^ was used based on random forest classifiers (RFCs). The ordinal classification was used because classes (degrees of impairment) are inherently ordered. Internally, the ordinal classifier comprised four binary RFCs which were trained to distinguish {0} vs {1, 2, 3, 4}, {0, 1} vs {2, 3, 4}, {0, 1, 2} vs {3, 4}, and {0, 1, 2, 3} vs {4}. Due to the class imbalance (see Table 1), we used the Synthetic Minority Oversampling Technique (SMOTE)^46^ to up-sample minority classes within each training fold. This ordinal classifier was trained and evaluated using 10-fold (stratified) cross-validation for each bradykinesia item. In addition, we also trained a (non-ordinal) binary RFC to distinguish between low ({0, 1}) and high ({2, 3, 4}) severity ratings.

### Composite bradykinesia score

For a given patient assessment, a composite bradykinesia score was obtained by summing the cross-validation predictions for the 10 individual model ratings (left and right ratings for each of the five items), giving a score on a scale of 0–40. This model composite score could then be compared to the clinical composite score, based on the ratings made by the clinical assessor.

We further examined how the composite bradykinesia scores varied with respect to the clinical ratings of MDS-UPDRS item 3.14 (known as global spontaneity of movement or body bradykinesia). Assessors are instructed that this rating should be based on the examiner’s global impression of bradykinesia symptoms after observing the patient for the entire assessment^3^.

Per-patient disagreement between the model and the composite bradykinesia score was further analysed by computing a proportion of residuals falling below a large clinically important difference (CID) threshold. Large CID was computed by applying the same percentage threshold as for the full UDPRS motor subscale estimated in the literature^25^. A value of 7.7 was calculated as a large CID for the composite bradykinesia score.

### Statistical analysis

The primary evaluation metric was the intraclass correlation (ICC), estimating the level of agreement of two raters (two-way random effects, absolute agreement, single rater ICC^47^), to measure agreement between the clinician and model estimate of composite bradykinesia score. This metric is widely used in interrater reliability and agreement analysis studies.

The secondary evaluation metrics were based on the individual item classifiers. For the MDS-UPDRS rating classifiers, we used balanced accuracy (the average recall obtained on each class) and acceptable accuracy (the proportion of estimates for which the residuals were zero or ±1). Balanced accuracy was chosen to account for the imbalanced dataset, while acceptable accuracy is chosen because it is not uncommon for MDS-UPDRS assessors to diverge from one another by one point^48^. For the binary classifiers, we used accuracy and the area under the curve of the receiver operator characteristic (AUC-ROC).

### Reporting summary

Further information on research design is available in the Nature Research Reporting Summary linked to this article.

## Supplementary information

Supplementary figures and methods

Reporting Summary

## Data Availability

The full data that support the findings of this study are not available for reasons of patient confidentiality and privacy. Anonymised summary data that support the findings of this study are available from the corresponding authors upon reasonable request.

## Code availability

Classification models were constructed using version 0.24.1 of scikit-learn (scikit-learn.org). Data processing, feature creation and statistical analysis used; version 1.3.5 of pandas (pandas.pydata.org), version 1.12.5 of numpy (numpy.org), version 1.7.3 of scipy (scipy.org) and version 0.5.1 of pingouin (pingouin-stats.org). Data visualisations were created using version 3.3.3 of matplotlib (matplotlib.org) and version 0.11.1 of seaborn (seaborn.pydata.org).
