# Supplementary material for: Computer vision quantification of whole-body Parkinsonian bradykinesia using a large multi-site population
Source: NPJ Parkinsons Dis. 2023 Jan 27;9:10. doi: 10.1038/s41531-023-00454-8 (PMC9883391; doi:10.1038/s41531-023-00454-8)
Supplement: Supplementary file 1 — Supplementary figures and methods [file 41531_2023_454_MOESM1_ESM.pdf]

Computer vision quantification of whole-body Parkinsonian bradykinesia using a large multi-site population

Gareth Morinan MSc[1,†], Yuriy Dushin MSc[1,†,*], Grzegorz Sarapata MSc[1], Samuel Rupprechter PhD[1], Yuwei Peng MSc[1], Christine Girges PhD[2], Maricel Salazar[2], Catherine Milabo[2], Krista Sibley MSc[2], Thomas Foltynie MD, PhD[2], Ioana Cociasu MD, PhD[3], Lucia Ricciardi MD, PhD[3], Fahd Baig MD, DPhil[3], Francesca Morgante MD, PhD[3,4], Louise-Ann Leyland PhD[5], Rimona S Weil MD, PhD[5], Ro'ee Gilron PhD[6], and Jonathan O'Keeffe MD, PhD[7,*]

[1]Machine Medicine Technologies Ltd., The Leather Market Unit 1.1.1 11/13 Weston Street, London SE1 3ER, UK
[2]Department of Clinical and Movement Neurosciences, Institute of Neurology, University College London, Queen Square, London WC1N 3BG, UK
[3]Neuroscience Research Centre, Molecular and Clinical Sciences Research Institute, St George's, University of London, Cranmer Terrace, London SW17 0RE, UK
[4]Department of Clinical and Experimental Medicine, University of Messina, Messina, Italy, Via Consolare Valeria, 98165, Messina, Italy
[5]Dementia Research Center, Institute of Neurology, University College London, Queen Square, London WC1N 3AR, UK
[6]The Starr Lab, University of California San Francisco, 513 Parnassus Ave, HSE-823, San Francisco, CA 94143, USA
[7]Machine Medicine Technologies Ltd., The Leather Market Unit 1.1.1 11/13 Weston Street, London SE1 3ER, UK
*Corresponding authors: yuriy, jonathan@machinemedicine.com
†These authors contributed equally to this work

Contents

- Supplementary Note 1: Individual classifier results
- Supplementary Note 2: Patient characteristics analysis
- Supplementary Note 3: Disease laterality analysis
- Supplementary Note 4: Height estimation model

Supplementary Note 1: Individual classifier results

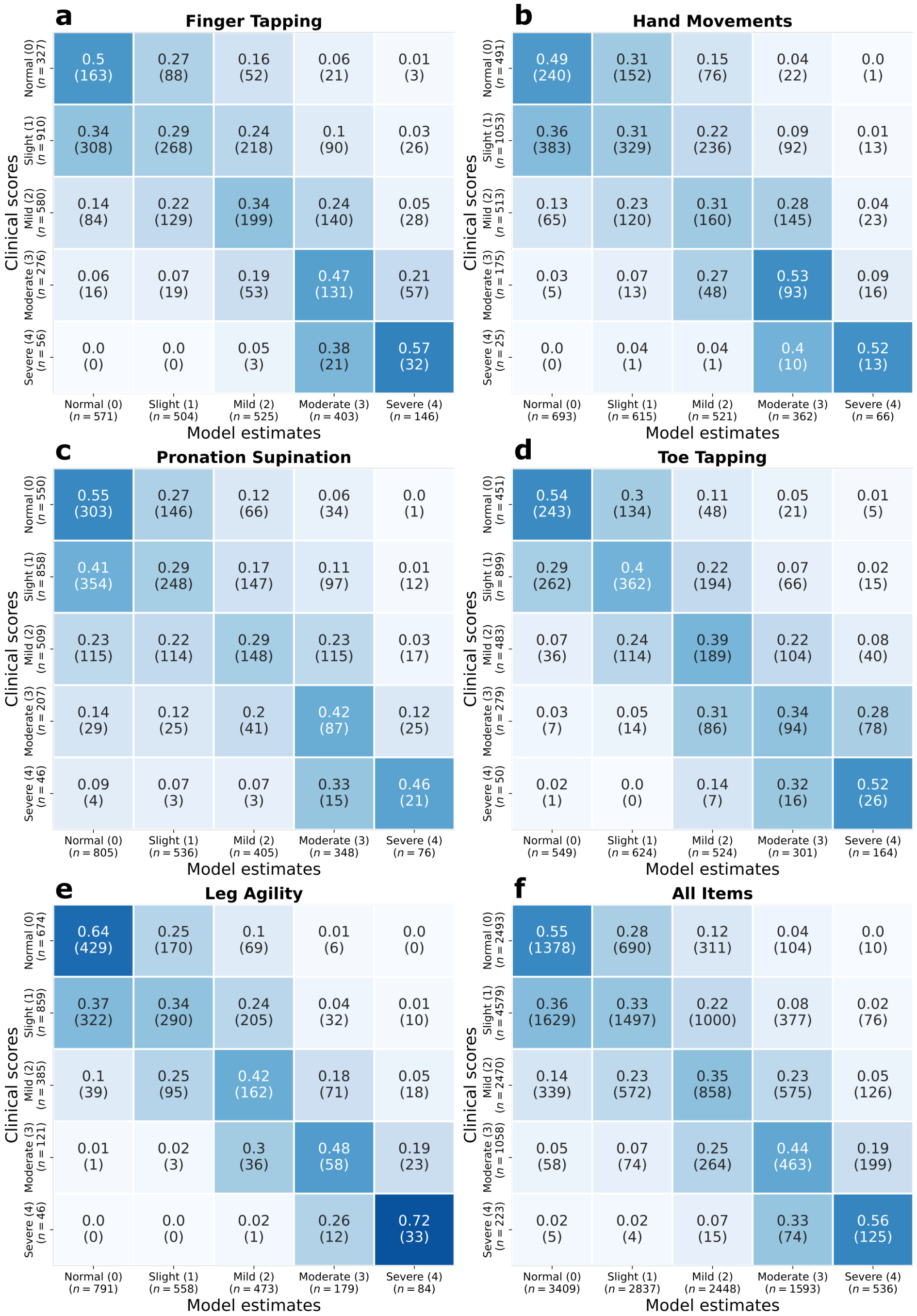

Supplementary Figure 1: The confusion matrices, between clinical ratings and model estimates, for each of the MDS-UPDRS classifiers; finger tapping (a), hand movements (b), pronation-supination (c), toe tapping (d), leg agility (e) and finally all items (f).

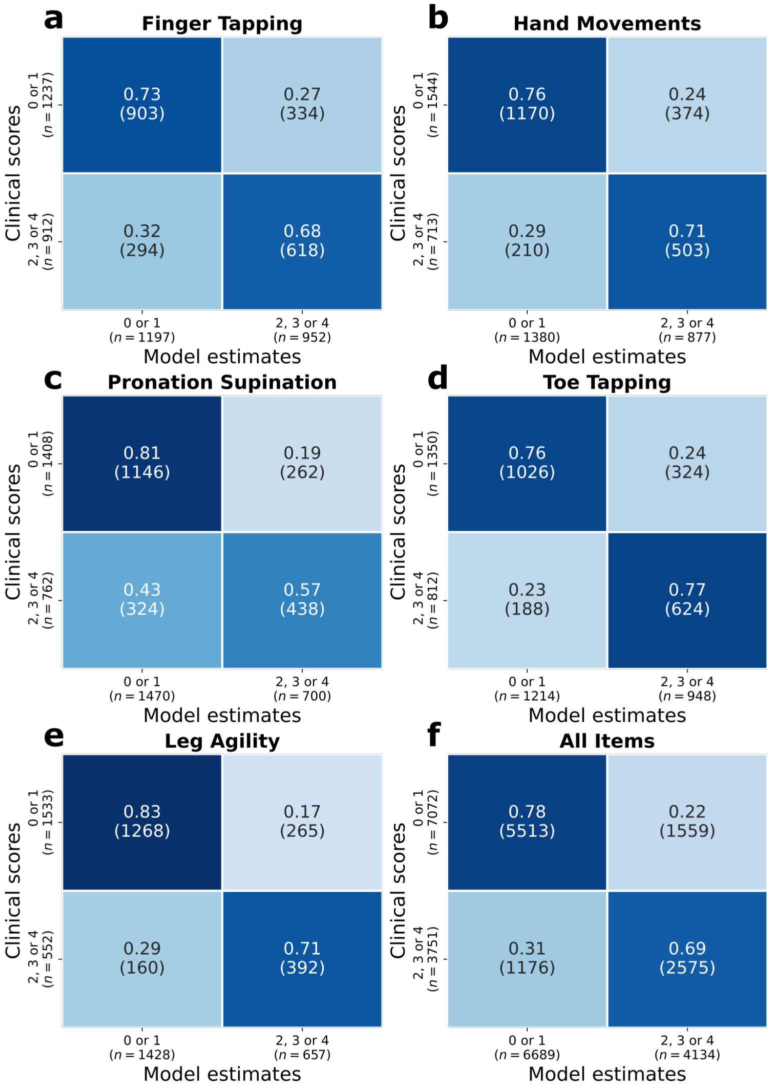

Supplementary Figure 2: The confusion matrices, between clinical ratings and model estimates, for each of the binary classifiers (ratings {0 or 1} vs {2, 3 or 4}); finger tapping (a), hand movements (b), pronation-supination (c), toe tapping (d), leg agility (e) and finally all items (f).

Supplementary Note 2: Patient characteristics analysis

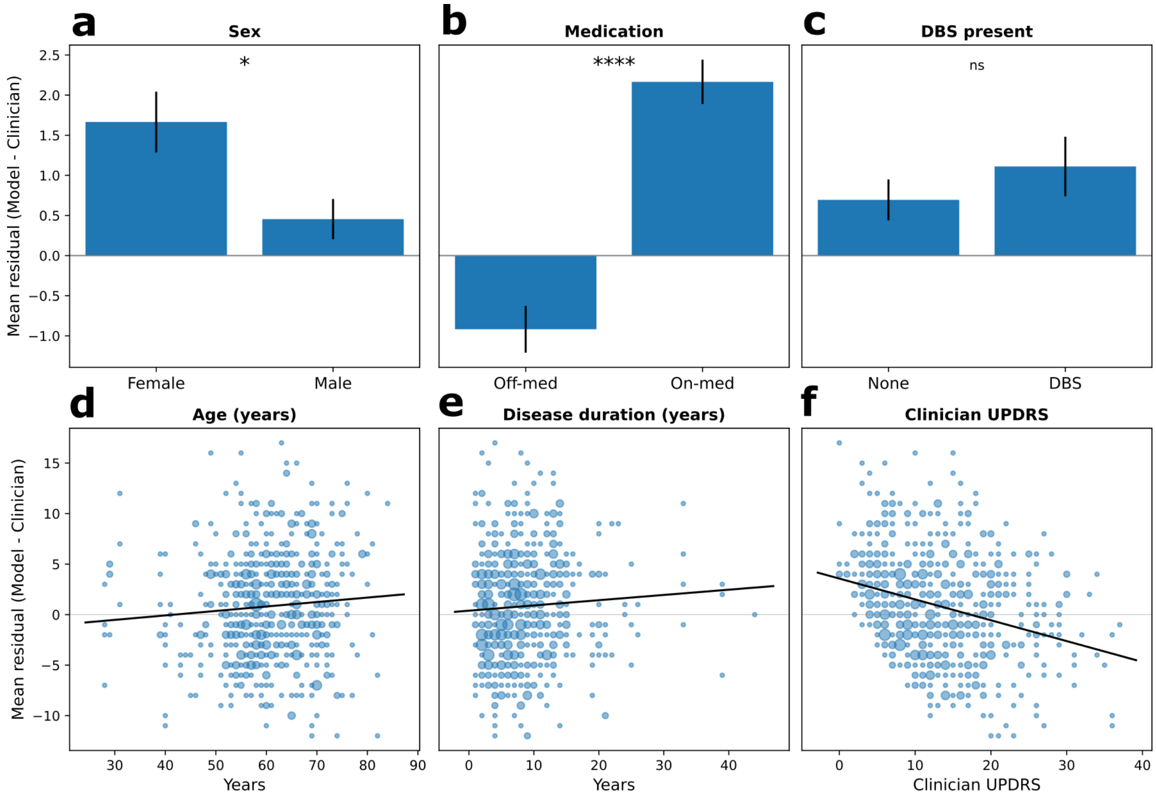

Supplementary Figure 3: The association between the residuals of the composite bradykinesia score (clinician score minus model score) and the additional patient characteristics that were available (see main manuscript for full breakdown). The difference in mean residual (error bars indicate standard error of the mean) for sex (a), medication status (b), and DBS surgery (c), as tested by a Mann-Whitney's U. This difference was significant for sex (p-value = 0.01), and for medication status (p-value < 0.0001), but not for whether the patient had undergone DBS surgery (p-value = 0.26). The correlation between the mean residual for patient age (d), disease duration (e), and clinician UPDRS (f), as tested by Pearson's r. This correlation was not significant for patient age (r = 0.08, p-value = 0.05), or for disease duration (r = 0.06, p-value = 0.14), but it was for disease severity (r = -0.29, p $<$ 0.0001). A significant correlation is to be expected for disease severity, given that MDS-UPDRS ratings are bounded (below by 0, above by 4); if a patient is given a clinical rating of 0, it is only possible for the model to match or over-estimate (it cannot under-estimate as this would mean estimating a value of -1), and vice versa for clinical ratings of 4. Similarly, the significant difference between the off- and on-medication assessments is also to be expected, as patients will exhibit significantly lower disease severity when on-medication. The significant difference in sex groups is unexpected; this could be explained by a systematic sex difference in the way in which MDS-UPDRS examination instructions are interpreted, or alternatively a difference in how these assessments are rated, but a larger dataset would be required to investigate this matter further.

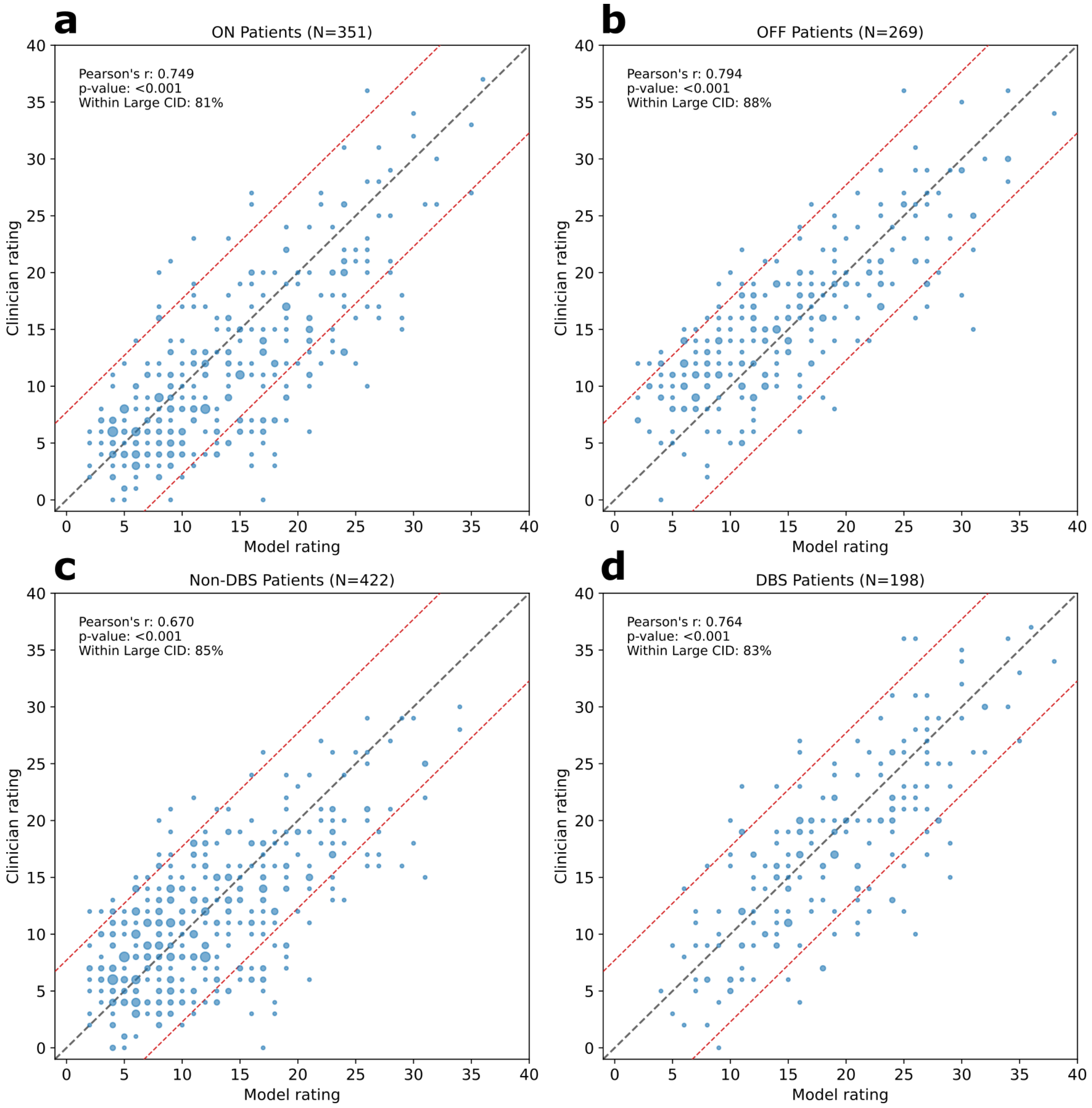

Supplementary Figure 4: Composite bradykinesia score (sum of items 3.4-3.8) estimation. (a, b) Scatterplots of composite bradykinesia scores for clinicians versus models for defined ON and OFF medication states. (c, d) Scatterplots of composite bradykinesia scores for clinicians versus models for assessments of patients with and with DBS surgery. The size of dots corresponds to the number of patients with that combination of clinician and model ratings. Red dashed lines indicate the large clinically important difference (CID) band.

Supplementary Note 3: Disease laterality analysis

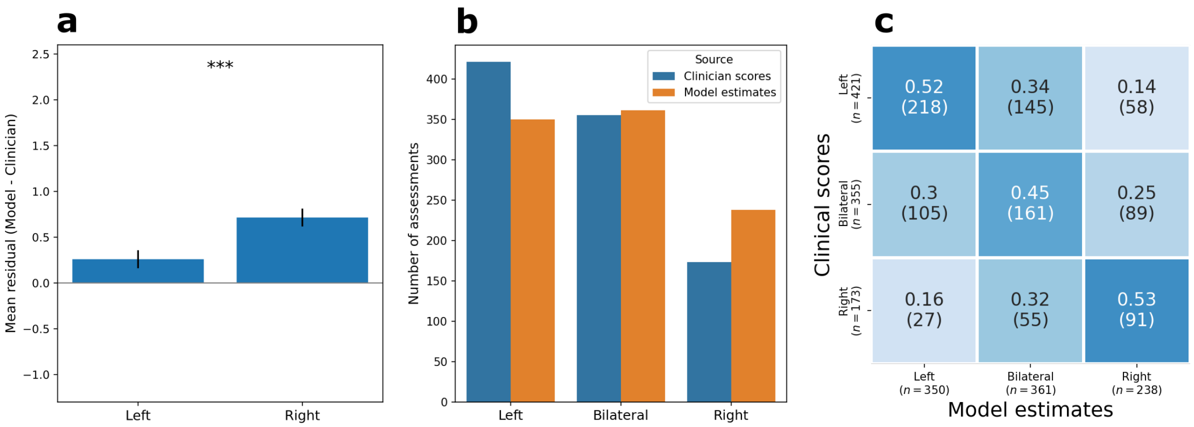

Supplementary Figure 5: The association between the composite bradykinesia score and laterality of the items and disease laterality. The laterality of items is defined through the sum of items on the corresponding side. Disease laterality is defined through a dominant side. A side is defined as dominant if the sum of scores on that side was greater than the opposite side by 2 or more [1]. If no side satisfies the criteria, the patient is defined as bilateral. (a) The difference in mean residual for left and right items (error bars indicate standard error of the mean), as tested by a Mann-Whitney's U. This difference was significant (p-value $<$ 0.001). Observed clinician scores were higher on the left (mean: 6.79, sem: 0.133) than the right (mean: 5.69, sem: 0.119) side. Model tendency to overestimate lower scores is a likely cause of the difference and not laterality itself. (b) Distribution of disease laterality in the assessments. Both the clinician and the model show prevalence of left-dominant disease over right. (c) Confusion matrix of agreement between clinician and model estimated disease laterality. The model was able to detect the laterality with a balanced accuracy of 50% (33% random chance accuracy).

Supplementary Note 4: Height estimation model

Model development

Signals from each patient were normalised by their estimated standing height in pixels. The height was estimated on a frame-by-frame basis using a linear model developed during previous research [2, 3]. This model was developed as follows.

OpenPose [4, 5] was used to extract 25 body key-points for the last frame of 356 "gait" videos (patient in full view, facing camera) and the first and last frame of the 322 "arising from chair" videos (i.e. the first frame in which patients were fully sitting and the last frame in which patients were fully standing). Frames of arising from chair videos were used as training data, and gait videos were used as validation data. These videos were recorded as part of MDS-UPDRS assessments (item 3.10 Gait and item 3.9 Arising From Chair) using the KELVIN™ platform [6].

The 300 possible distances between any two body key-points were computed for each frame. These distances (i.e. the features) were then ranked and included in a series of linear regression models. First, we divided each feature by the true body height and computed the variation of these values across all frames. Features were then ranked from lowest to highest variation, based on the idea that features with high variability would likely be less useful for prediction (e.g. the distance from left toe to right shoulder is vastly different depending on whether a patient is sitting or standing). Second, we computed the correlation of each feature with true body height and ranked them from highest to lowest correlation coefficient. This was based on the idea that the most predictive features should have a high correlation with body height.

For each of these two rankings we estimated 300 linear regression models. Starting with a model including only the "best" (highest ranked) feature, we then repeatedly added the next highest ranked feature. For each model we computed the mean absolute error on both training and validation data. Including additional features will always decrease the training error, but validation error will only decrease in the beginning and at some point start to increase as the model starts to over-fit. Through visual inspection of the training and validation error curves (Supplementary Figure 6) the point at which validation error was minimal was chosen. Twenty-seven features were selected from each approach.

For the final set of features we set two conditions: (a) Only features selected by both feature-ranking approaches were selected for the final classifier. (b) Only features for which both left and right version were selected were included in the final classifier (e.g. if right knee to right toe was included, left knee to left toes also needed to be included).

In the final classifier we also included torso length ("mid-hip" to "neck" key-points) as a feature even though it was not selected by both approaches. This was done because an earlier model had also included that distance, and we felt the feature might have been unfairly penalized because patients were often already leaning forward at the start of the video as they were preparing to arise from the chair. The final model included the following distances: Neck-RShoulder, Neck-LShoulder, MidHip-RHip, MidHip-LHip, Neck-MidHip, RKnee-RAnkle, LKnee-LAnkle.

The model was estimated on the full training set and then coefficients were averaged across left and right side, i.e. `featureR` (e.g. "neck to right shoulder") and `featureL` ("neck to left shoulder") were both set to the value (`featureR` + `featureL`) / 2.

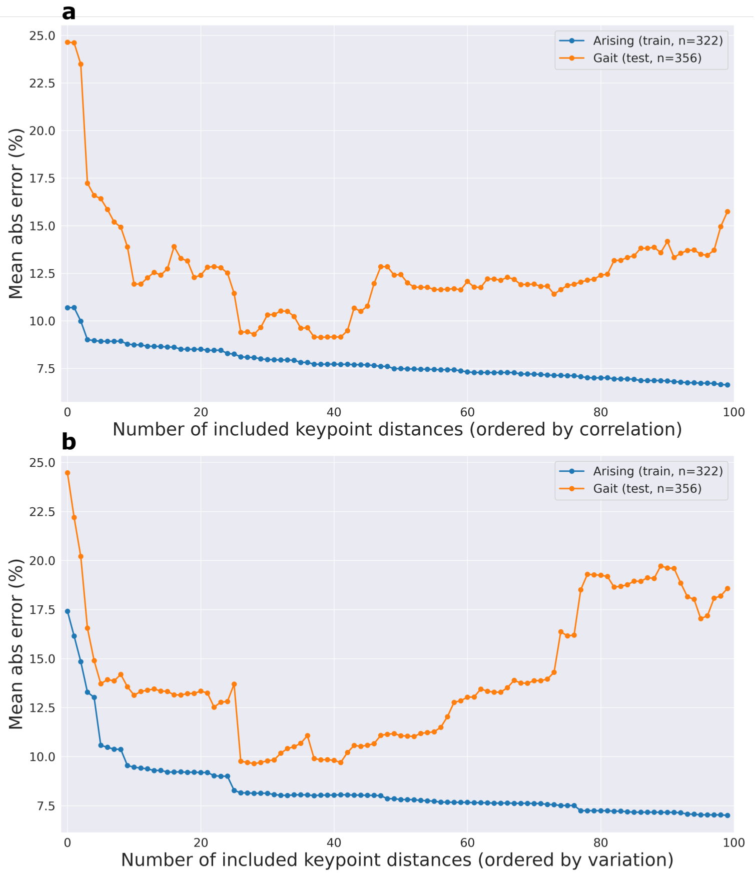

Supplementary Figure 6: Summary of height estimation model performance. Training ($n = 322$) and test ($n = 356$) set performance (mean absolute error across videos) during the first 100 step-wise inclusion of distances ranked by correlation (a) or variation (b).

Smoothing and confidence weighting

Height estimation was smoothed across frames within a video by replacing the estimation at each frame with the mean estimation of the 15 previous frames.

In addition to the coordinates of each key-point, OpenPose also estimates a confidence score between 0 and 1. We calculate the confidence for each distance as the minimum of the confidence scores of its two endpoints and included this score in the height estimation model. Before passing the distances to the linear regression model, each distance x_i was re-estimated as

$$x_i \leftarrow c_i \times x_i + (1 - c_i) \times z_i, \tag{1}$$

where c_i is the confidence of distance i and z_i is the prediction of x_i using all the other distances $x_{j \neq i}$. This prediction was estimated as

$$z_i = \frac{1}{\sum_j r_j} \sum_j x_j^{(i)} \times w_j^{(i)} \times r_{i,j}, \tag{2}$$

where $r_{i,j}$ is the Pearson correlation coefficient between distances i and j, and $w_j^{(i)}$ is the coefficient of the linear prediction of x_i using x_j, so that $x_i \approx x_j \times w_j^{(i)}$. The correlation coefficients r were estimated across a large number of frames and videos and are set as constant within the model, while the coefficients w are estimated on a per-video basis as the average prediction across all frames, meaning

$$\mathbf{x}_i = \mathbf{x}_j w_j + \epsilon, \tag{3}$$

where $\mathbf{x}_i$ and $\mathbf{x}_j$ are vectors containing all the distances x_i and x_j within a video (one distance x per frame).

References

[1] Elkurd, M., Wang, J. & Dewey, R. B. Lateralization of motor signs affects symptom progression in parkinson disease. *Frontiers in Neurology* **12** (2021). URL https://www.frontiersin.org/articles/10.3389/fneur.2021.711045.

[2] Morinan, G. *et al.* Computer-vision based method for quantifying rising from chair in parkinson's disease patients. *Intelligence-Based Medicine* **6**, 100046 (2022).

[3] Rupprechter, S. *et al.* A clinically interpretable computer-vision based method for quantifying gait in parkinson's disease. *Sensors* **21**, 5437 (2021).

[4] Cao, Z., Simon, T., Wei, S.-E. & Sheikh, Y. Realtime multi-person 2d pose estimation using part affinity fields. In *CVPR* (2017).

[5] Simon, T., Joo, H., Matthews, I. & Sheikh, Y. Hand keypoint detection in single images using multiview bootstrapping. In *CVPR* (2017).

[6] Machine Medicine Technologies Limited. The company's webplatform. https://kelvin.machinemedicine.com/ (2021).
