# Supplementary material for: Computer vision quantification of whole-body Parkinsonian bradykinesia using a large multi-site population
Source: NPJ Parkinsons Dis. 2023 Jan 27;9:10. doi: 10.1038/s41531-023-00454-8 (PMC9883391; doi:10.1038/s41531-023-00454-8)
Supplement: Supplementary file 2 — Reporting Summary [file 41531_2023_454_MOESM2_ESM.pdf]

nature portfolio

Corresponding author(s): Yuriy Dushin (yuriy@machinemedicine.com)

Last updated by author(s): Dec 1, 2022

Reporting Summary

Nature Portfolio wishes to improve the reproducibility of the work that we publish. This form provides structure for consistency and transparency in reporting. For further information on Nature Portfolio policies, see our Editorial Policies and the Editorial Policy Checklist.

Statistics

For all statistical analyses, confirm that the following items are present in the figure legend, table legend, main text, or Methods section.

n/a	Confirmed	
☐	☒	The exact sample size (*n*) for each experimental group/condition, given as a discrete number and unit of measurement
☐	☒	A statement on whether measurements were taken from distinct samples or whether the same sample was measured repeatedly
☐	☒	The statistical test(s) used AND whether they are one- or two-sided<br>*Only common tests should be described solely by name; describe more complex techniques in the Methods section.*
☒	☐	A description of all covariates tested
☒	☐	A description of any assumptions or corrections, such as tests of normality and adjustment for multiple comparisons
☐	☒	A full description of the statistical parameters including central tendency (e.g. means) or other basic estimates (e.g. regression coefficient) AND variation (e.g. standard deviation) or associated estimates of uncertainty (e.g. confidence intervals)
☐	☒	For null hypothesis testing, the test statistic (e.g. *F*, *t*, *r*) with confidence intervals, effect sizes, degrees of freedom and *P* value noted<br>*Give P values as exact values whenever suitable.*
☒	☐	For Bayesian analysis, information on the choice of priors and Markov chain Monte Carlo settings
☒	☐	For hierarchical and complex designs, identification of the appropriate level for tests and full reporting of outcomes
☐	☒	Estimates of effect sizes (e.g. Cohen's *d*, Pearson's *r*), indicating how they were calculated

Our web collection on statistics for biologists contains articles on many of the points above.

Software and code

Policy information about availability of computer code

Data collection	version 1.3.5 of pandas (pandas.pydata. org), version 1.12.5 of numpy (numpy.org), version 1.7.3 of scipy (scipy.org)
Data analysis	Classification models were constructed using version 0.24.1 of scikit-learn (scikit-learn.org). Feature creation and statistical analysis used; version 1.3.5 of pandas (pandas.pydata. org), version 1.12.5 of numpy (numpy.org), version 1.7.3 of scipy (scipy.org) and version 0.5.1 of pingouin (pingouin-stats.org). Data visualisations were created using version 3.3.3 of matplotlib (matplotlib.org) and version 0.11.1 of seaborn (seaborn.pydata.org).

For manuscripts utilizing custom algorithms or software that are central to the research but not yet described in published literature, software must be made available to editors and reviewers. We strongly encourage code deposition in a community repository (e.g. GitHub). See the Nature Portfolio guidelines for submitting code & software for further information.

Data

Policy information about availability of data

All manuscripts must include a data availability statement. This statement should provide the following information, where applicable:

- Accession codes, unique identifiers, or web links for publicly available datasets
- A description of any restrictions on data availability
- For clinical datasets or third party data, please ensure that the statement adheres to our policy

The full data that support the findings of this study are not available for reasons of patient confidentiality and privacy. Anonymised summary data that support the findings of this study are available from the corresponding authors upon reasonable request.

Human research participants

Policy information about studies involving human research participants and Sex and Gender in Research.

Reporting on sex and gender	Analysis is performed independent of gender or sex. This information is available for a subset of participants and was self reported. Sub analysis of gender subgroups was performed to estimate if there is a significant difference in the performance of proposed system between genders. Data is available for 191 Females and 429 Males.
Population characteristics	Age, gender, disease duration, medication status, deep brain stimulation status.
Recruitment	No recruitment was conducted. Data was collected as a part of routine clinical assessments. No enrollment criteria was applied and all available data taken.
Ethics oversight	Data was collected according to the procedures and oversight of the following institutions: Department of Clinical and Movement Neurosciences, Institute of Neurology, University College London;<br>Neuroscience Research Centre, Molecular and Clinical Sciences Research Institute, St. George's, University of London;<br>Dementia Research Center, Institute of Neurology, University College London;<br>Parkinson's Disease and Movement Disorders Center, Baylor College of Medicine;<br>The Starr Lab, University of California San Francisco.

Note that full information on the approval of the study protocol must also be provided in the manuscript.

Field-specific reporting

Please select the one below that is the best fit for your research. If you are not sure, read the appropriate sections before making your selection.

☒ Life sciences ☐ Behavioural & social sciences ☐ Ecological, evolutionary & environmental sciences

For a reference copy of the document with all sections, see nature.com/documents/nr-reporting-summary-flat.pdf

Life sciences study design

All studies must disclose on these points even when the disclosure is negative.

Sample size	This is observational, non-hypothesis testing study. Sample size is determined by the data availability. All available data was taken.
Data exclusions	No data were excluded.
Replication	Code and data versioning was implemented with executed analysis results saved. The analysis was repeated and results replicated.
Randomization	Not relevant, there are no group allocation in this study. Residual analysis was performed for population subgroups to validate the results for different populations.
Blinding	No blinding performed. Results of the algorithms were cross validated to account for overfitting.

Reporting for specific materials, systems and methods

We require information from authors about some types of materials, experimental systems and methods used in many studies. Here, indicate whether each material, system or method listed is relevant to your study. If you are not sure if a list item applies to your research, read the appropriate section before selecting a response.

Materials & experimental systems

n/a	Involved in the study
☒	☐ Antibodies
☒	☐ Eukaryotic cell lines
☒	☐ Palaeontology and archaeology
☒	☐ Animals and other organisms
☐	☒ Clinical data
☒	☐ Dual use research of concern

Methods

n/a	Involved in the study
☒	☐ ChIP-seq
☒	☐ Flow cytometry
☒	☐ MRI-based neuroimaging

Clinical data

Policy information about clinical studies

All manuscripts should comply with the ICMJE guidelines for publication of clinical research and a completed CONSORT checklist must be included with all submissions.

Clinical trial registration: NCT01971242, NCT03652870, NCT03582891

Study protocol: Protocols can obtained through a relevant contact available in clinicaltrials.gov

Data collection: This research is using data collected as a part of routine clinical assessments in London, UK and San Francisco, USA over period Nov 2018- Sep 2021

Outcomes: N/A, this research used the data collected as a part of a clinical trial, but
